# Signal amplification and optimization of riboswitch-based hybrid inputs by modular and titratable toehold switches

**DOI:** 10.1186/s13036-021-00261-w

**Published:** 2021-03-19

**Authors:** Yunhee Hwang, Seong Gyeong Kim, Sungho Jang, Jongmin Kim, Gyoo Yeol Jung

**Affiliations:** 1 Department of Chemical Engineering, Pohang University of Science and Technology, 77 Cheongam-ro, Nam-gu, Pohang, Gyeongbuk 37673 South Korea; 2 Department of Bioengineering and Nano-Bioengineering, Incheon National University, 119 Academy-ro, Yeonsu-gu, Incheon, 22012 South Korea; 3 Division of Bioengineering, College of Life Sciences and Bioengineering, Incheon National University, 119 Academy-ro, Yeonsu-gu, Incheon, 22012 South Korea; 4 Division of Integrative Biosciences and Biotechnology, Pohang University of Science and Technology, 77 Cheongam-ro, Nam-gu, Pohang, Gyeongbuk 37673 South Korea; 5 School of Interdisciplinary Bioscience and Bioengineering, Pohang University of Science and Technology, 77 Cheongam-ro, Nam-gu, Pohang, Gyeongbuk 37673 South Korea

**Keywords:** Biosensor, Riboswitch, Coenzyme B_12_, Genetic circuit, Toehold switch

## Abstract

**Background:**

Synthetic biological circuits are widely utilized to control microbial cell functions. Natural and synthetic riboswitches are attractive sensor modules for use in synthetic biology applications. However, tuning the fold-change of riboswitch circuits is challenging because a deep understanding of the riboswitch mechanism and screening of mutant libraries is generally required. Therefore, novel molecular parts and strategies for straightforward tuning of the fold-change of riboswitch circuits are needed.

**Results:**

In this study, we devised a toehold switch-based modulator approach that combines a hybrid input construct consisting of a riboswitch and transcriptional repressor and *de-novo-*designed riboregulators named toehold switches. First, the introduction of a pair of toehold switches and triggers as a downstream signal-processing module to the hybrid input for coenzyme B_12_ resulted in a functional riboswitch circuit. Next, several optimization strategies that focused on balancing the expression levels of the RNA components greatly improved the fold-change from 260- to 887-fold depending on the promoter and host strain. Further characterizations confirmed low leakiness and high orthogonality of five toehold switch pairs, indicating the broad applicability of this strategy to riboswitch tuning.

**Conclusions:**

The toehold switch-based modulator substantially improved the fold-change compared to the previous sensors with only the hybrid input construct. The programmable RNA-RNA interactions amenable to in silico design and optimization can facilitate further development of RNA-based genetic modulators for flexible tuning of riboswitch circuitry and synthetic biosensors.

**Supplementary Information:**

The online version contains supplementary material available at 10.1186/s13036-021-00261-w.

## Background

Synthetic biology is an emerging engineering discipline that aims to design and build biological parts, devices, and systems based on the understanding of biological systems [1]. One important research area in synthetic biology is to embed synthetic biological circuits in microbial cells to control their responses to environmental inputs [2]. Simple genetic parts are assembled to construct complex genetic circuits with useful functions. Numerous applications utilizing genetic circuits have been reported, such as monitoring of small molecules, control of metabolic pathways, directed evolution of enzymes, and logic computation [3–6].

A riboswitch is an RNA-based, *cis*-acting regulator that controls the expression of the gene in the same mRNA where the riboswitch is encoded [7, 8]. The riboswitch is composed of an aptamer domain capable of binding to a ligand and an expression platform that undergoes structural changes in response to ligand binding to the aptamer. Riboswitches have been utilized as input parts to construct synthetic genetic circuits for a variety of applications by monitoring intracellular metabolite concentrations and in turn regulating the expression of functional genes. For instance, the metabolism of vitamin B_12_ was monitored by measuring the intracellular adenosylcobalamin (AdoCbl) concentration using an AdoCbl-responsive riboswitch [9]. A synthetic L-lysine riboswitch was utilized for dynamic control of the lysine transport system in lysine-producing strain and a natural lysine riboswitch with *tetA* selection marker gene was used for L-lysine pathway optimization [10, 11]. Also, artificial L-tryptophan riboswitch was utilized for high-throughput screening and selection of mutant library [12]. Finally, the enzymatic activity of caffeine demethylase was improved and an acid-tolerant phenotype was obtained through directed evolution with theophylline riboswitch and pH-responsive riboswitch, respectively [13, 14].

Analogous to other biosensors, the fold-change is key parameter of riboswitch performance [15, 16]. The fold-change refers to the ratio of the minimum and maximum output signals. For synthetic genetic circuits, specific and robust gene expression regulation is highly desirable to maximize the regulatory outcome while minimizing leaky expression that can contribute to gene expression noise and unnecessary consumption of cellular resources [17]. Therefore, a fold-change of riboswitch should be as high as possible to distinguish the output signals according to varying metabolite concentrations clearly.

Previous riboswitch engineering studies have mostly focused on the direct modification of the riboswitch itself, namely, the aptamer domain or expression platform [18, 19]. However, the fold-change was marginally improved, and a detailed understanding of the structure, biochemistry, and evolution steps of a target riboswitch was required for this strategy. Also, an indirect method modulated the copy number of a riboswitch molecule using promoter variants or different plasmids [20], resulting in a minor improvement in fold-changes.

An alternative approach for parameter tuning is to introduce new genetic regulation modules. Through this strategy, the parameters of the riboswitch circuit can be adjusted beyond the limit of natural riboswitches through multi-step binding events in the added regulatory modules. For example, we previously reported a hybrid input riboswitch circuit that combined a natural riboswitch and transcriptional repressors [21]. The hybrid input inverted the output signal from the riboswitch and amplified its fold-change from 7.5- to 32.1-fold without extensive characterization or direct modifications of the riboswitch. However, the fold-change was still small compared to optimized transcription factor-based circuits that can reach up to several hundred-fold. Further improvement of the fold-change with this strategy would require the incorporation of signal-amplifying modules, where the performance of the resulting riboswitch circuit is determined by the properties of these additional modules.

Progress in RNA synthetic biology has provided a multitude of readily usable novel parts that may be integrated with existing synthetic circuit designs. We focused on a new type of RNA-based regulator known as a toehold switch, which provides a library of *de-novo*-designed regulatory parts with high fold-change and orthogonality [22]. The ribosome binding site of a toehold switch is exposed upon specific binding to a cognate trigger RNA, allowing for precise control of gene expression at the post-transcription level [22, 23]. We hypothesized that the toehold switch can be used to adjust the fold-change of the riboswitch circuits by inserting another signal propagation stage. The toehold switch would allow for flexible tuning of the riboswitch circuits by independently modulating the expression levels of the trigger and switch RNA. Additionally, the large fold-change of toehold switches may further amplify the output signal from the riboswitch circuits.

In this study, we showed that the toehold switch can be utilized to modulate the properties of a riboswitch-based sensor. Previously reported hybrid input parts for coenzyme B_12_ [21] were combined with toehold switch-trigger pairs. The resulting circuits showed high orthogonality, low leakiness, and substantial improvement in fold-change. These results demonstrate that toehold switches can provide programmable and modular plug-and-play genetic parts for the response tuning of riboswitch circuitry.

## Results and discussion

### Construction of a toehold switch-based modulator

**Fig. 1:**
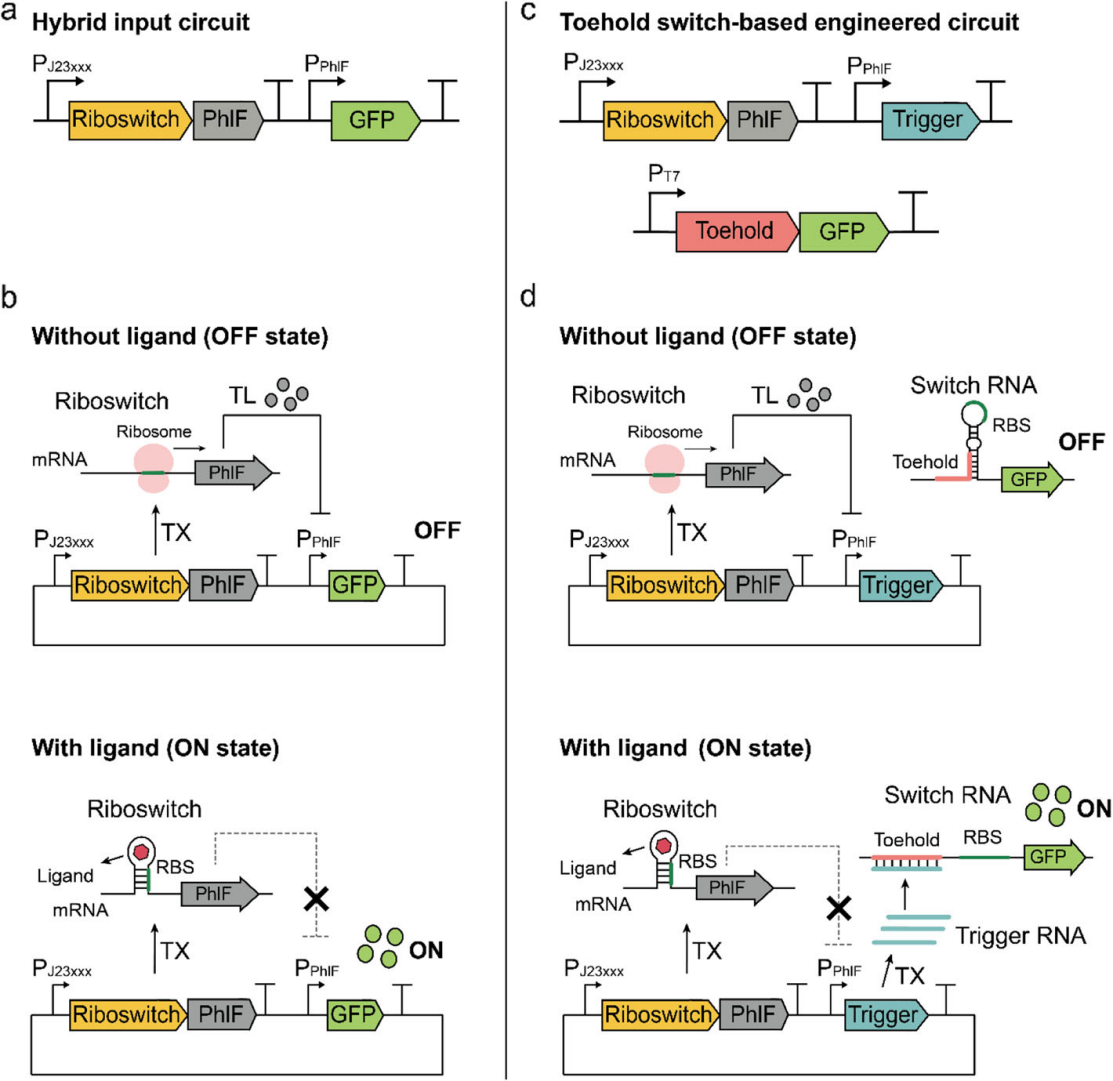
Overall scheme of the hybrid input circuit and toehold switch-based modulator. **a** A previously characterized hybrid input riboswitch circuit combined an off-type coenzyme B_12_ riboswitch with transcriptional repressors to invert the output signal [21]. **b** ON/OFF modes of the hybrid input circuit. In the absence of a ligand, the transcriptional repressor is expressed by the off-type coenzyme B_12_ riboswitch and represses the transcription of GFP (green fluorescence protein). In the presence of a ligand, expression of the transcriptional repressor is inhibited, resulting in transcription of GFP. **c** Scheme of the toehold switch-based modulator circuit. The riboswitch controlled the expression of transcriptional repressors; however, in this study, the transcriptional repressors regulated the expression of trigger RNAs, rather than reporter proteins. Then, the interaction between the toehold switch and trigger RNA activates the expression of the reporter gene. **d** ON/OFF modes of the toehold switch-based modulator circuit. In the absence of a ligand, the riboswitch and transcriptional repressor is transcribed by the constitutive promoter. Since the riboswitch is off type, the translation of the transcriptional repressor is allowed, which in turn represses the transcription of trigger RNA. Consequently, the toehold switch RNA maintains the translation-repressing hairpin structure, occluding the ribosomal binding site (RBS) of the reporter gene. On the contrary, in the presence of a ligand, expression of the transcriptional repressor is inhibited, resulting in robust transcription of trigger RNAs. The binding of the trigger RNA to the cognate toehold switch RNA exposes the RBS to activate translation of the reporter gene. The P_J23xxx_, P_PhlF_, P_T7_ refer to the Anderson promoter series, PhlF transcriptional repressor cognate promoter, and T7 promoter, respectively

First, we evaluated toehold switches as modular plug-and-play genetic parts in the riboswitch circuitry. The base circuit to be engineered was a hybrid input riboswitch circuit built using an off-type coenzyme B_12_ riboswitch and transcriptional repressors (Fig. 1a) [21]. The coenzyme B_12_ riboswitch is derived from 5′ untranslated region of *cbiA* from *Salmonella typhimurium* [24], and transcriptional repressors are TetR homologs with strong repressibility and high orthogonality [25]. Binding of the coenzyme B_12_ to the riboswitch downregulates the expression of the transcriptional repressor, which in turn activates the expression of the final reporter gene under the control of the transcriptional repressor (Fig. 1b). Previously, several combinations of constitutive promoters and transcriptional repressors were tested to modulate the fold-change of the riboswitch circuits. Among the tested combinations, the P100 circuit composed of the BBa_J23100 promoter and PhlF transcriptional repressor showed the highest fold-change of 32.1 [21].

We used P100 as the target riboswitch circuit to be engineered owing to its highest fold-change. In the engineered circuit, the direct regulation of the PhlF transcriptional repressor on its cognate promoter driving green fluorescent protein (GFP) expression was modified to an indirect regulation by introducing toehold switch-trigger pairs (Fig. 1c). Here, the transcriptional repressor regulates the expression of the trigger RNA, not the expression of the reporter gene (Fig. 1d). Then, the trigger RNA specifically recognizes its cognate toehold switch and forms RNA-RNA interactions, exposing the ribosomal binding site (RBS) to activate the translation of the reporter gene (Figure S1).

### Toehold switch-based modulator optimization by adjusting expression levels of switch RNA and trigger RNA

**Fig. 2:**
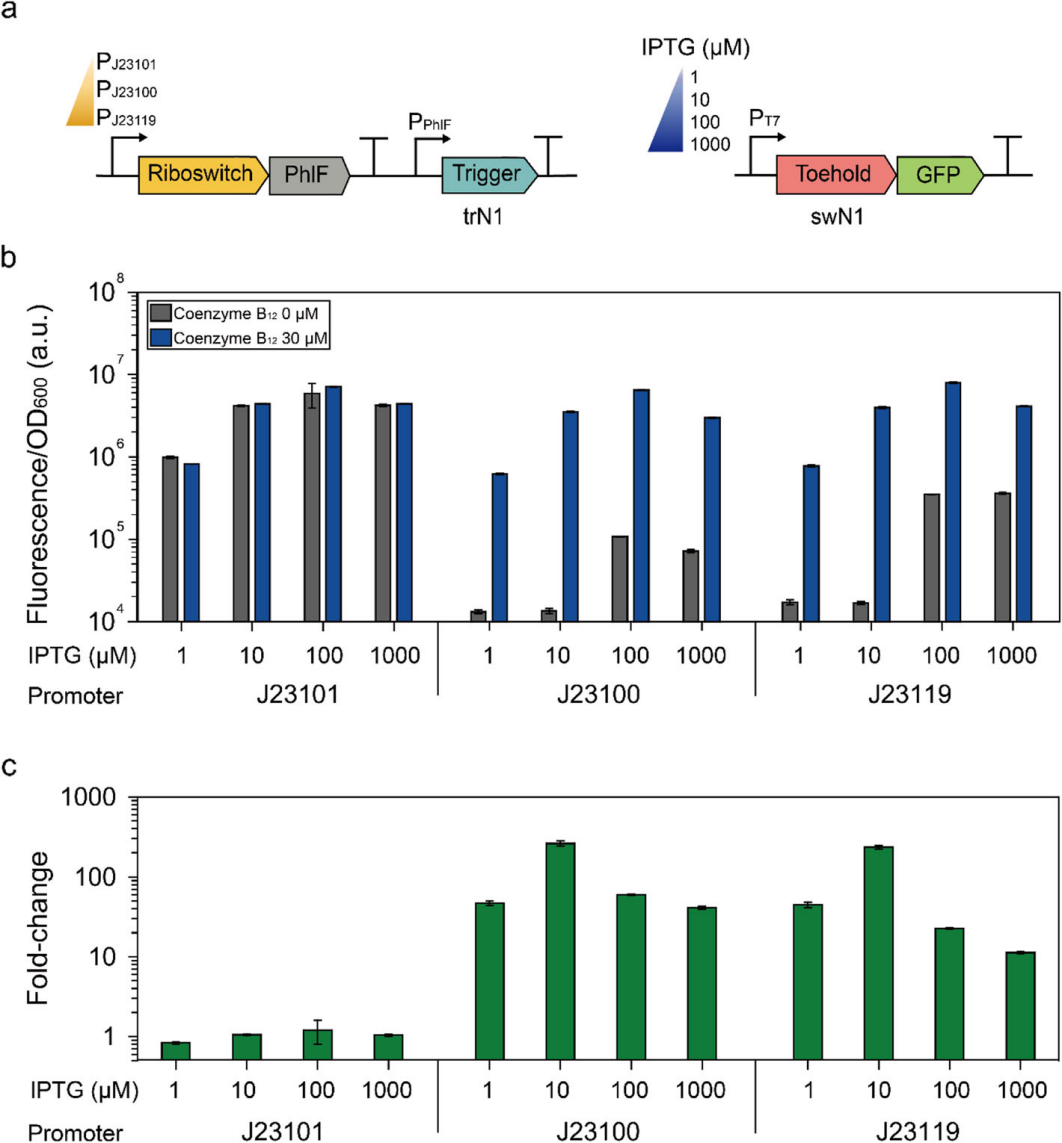
Optimization of the toehold switch-based modulators with various promoters and IPTG concentration. **a** Optimization strategy. Three constitutive promoters – the Anderson promoter collection BBa_J23101 (Relative strength: 0.7), BBa_J23100 (Relative strength: 1) and BBa_J23119 (Relative strength: > 1) –were used and different concentrations of IPTG (1, 10, 100, 1000 μM) were added. The relative strength data is from the Anderson promoter collection (http://parts.igem.org/Promoters/Catalog/Anderson). **b** Fluorescence measurements with or without coenzyme B_12_ (30 μM) for trN1-swN1 and modified trN1-swN1 strains in which the promoter for the riboswitch was changed to J23101 or J23119. **c** Fold-change of GFP expression levels for the strains used in (**b**). Error bars indicate standard deviations from biological triplicate measurements

We selected an AND-computing toehold switch (ACTS_TypeII_N1) that showed the highest fold-change among reported trigger-toehold switch pairs [23] for engineering the riboswitch circuit. The toehold switch was inserted into the P100 circuit (Fig. 2a). In the engineered circuit, the PhlF-cognate promoter drives the expression of trigger RNA (trN1), which hybridizes to its cognate toehold switch (swN1) to activate *gfp* gene expression. The engineered circuit was transformed into the *Escherichia coli* BL21 Star (DE3) strain, which is RNase-deficient, to confirm the functionality of the circuit. Since the toehold switch-based modulator showed the highest fluorescence when coenzyme B_12_ was added at 30 μM (Figure S2a, b), 0 and 30 μM coenzyme B_12_ were used to evaluate the fold-change of the circuits. As a result, the engineered circuit trN1-swN1, which has constitutive promoter BBa_J23100 to transcribe riboswitch and PhlF, effectively activated the gene expression by 59.9-fold when 30 μM coenzyme B_12_ and 100 μM isopropyl β-D-1-thiogalactopyranoside (IPTG) were added (Fig. 2b, c). Compared to the P100 circuit, the engineered circuit showed a 1.86-fold increase in fold-change, indicating the potential of the toehold switch as a modular genetic part for signal amplification.

The RNA-RNA interaction of trigger and switch molecules is the key feature in the toehold switch-based modulator. The balanced expression of the RNA molecules is important for achieving the maximum regulatory outcome. Therefore, we hypothesized that additional signal amplification could be achieved by adjusting the expression levels of the trigger RNA and the switch RNA. First, two additional constitutive promoters, BBa_J23101 and BBa_J23119, were introduced (Fig. 2a). The amount of transcripts for PhlF can be adjusted through the change in promoter strength, which in turn can control the expression level of trigger RNA. Second, the expression level of the switch RNA was controlled with different IPTG concentrations (Fig. 2a). The overexpression of switch RNA would increase the basal expression of the GFP reporter, while very low expression of switch RNA would lower the overall performance of the genetic circuit. The fluorescence values from the circuit variants were measured with or without coenzyme B_12_ (30 μM) (Fig. 2b). As a result, the highest fold-change of 261-fold was achieved using promoter BBa_J23100 and 10 μM IPTG (Fig. 2b, c). Notably, the modular architecture of the toehold switch pair allowed for independent modulation of the expression levels of each interacting part, resulting in efficient optimization of the circuit performance.

It is evident that an imbalance in the expression levels of trigger RNA and switch RNA generally results in sub-optimal circuit performance. For example, high fluorescence values were observed in the absence of coenzyme B_12_ for the circuits with the BBa_J23101 promoter. This background expression might have originated from the high basal expression of the trigger RNA due to the insufficient PhlF expression with the low transcriptional efficiency of BBa_J23101. In contrast, overexpression of the switch RNA with 1000 μM IPTG resulted in decreased fluorescence intensity in the presence of coenzyme B_12_ compared to that with 100 μM IPTG because of the burden of gene expression [26, 27]. The dose-response curves and half-maximal effective concentrations (EC_50_) for each circuit are shown in Figure S2. Overall, the properties of the engineered circuit with a toehold switch were optimized by balancing the expression levels of trigger and switch RNA.

### Scalability, orthogonality, and compatibility of toehold switch-based modulators

**Fig. 3:**
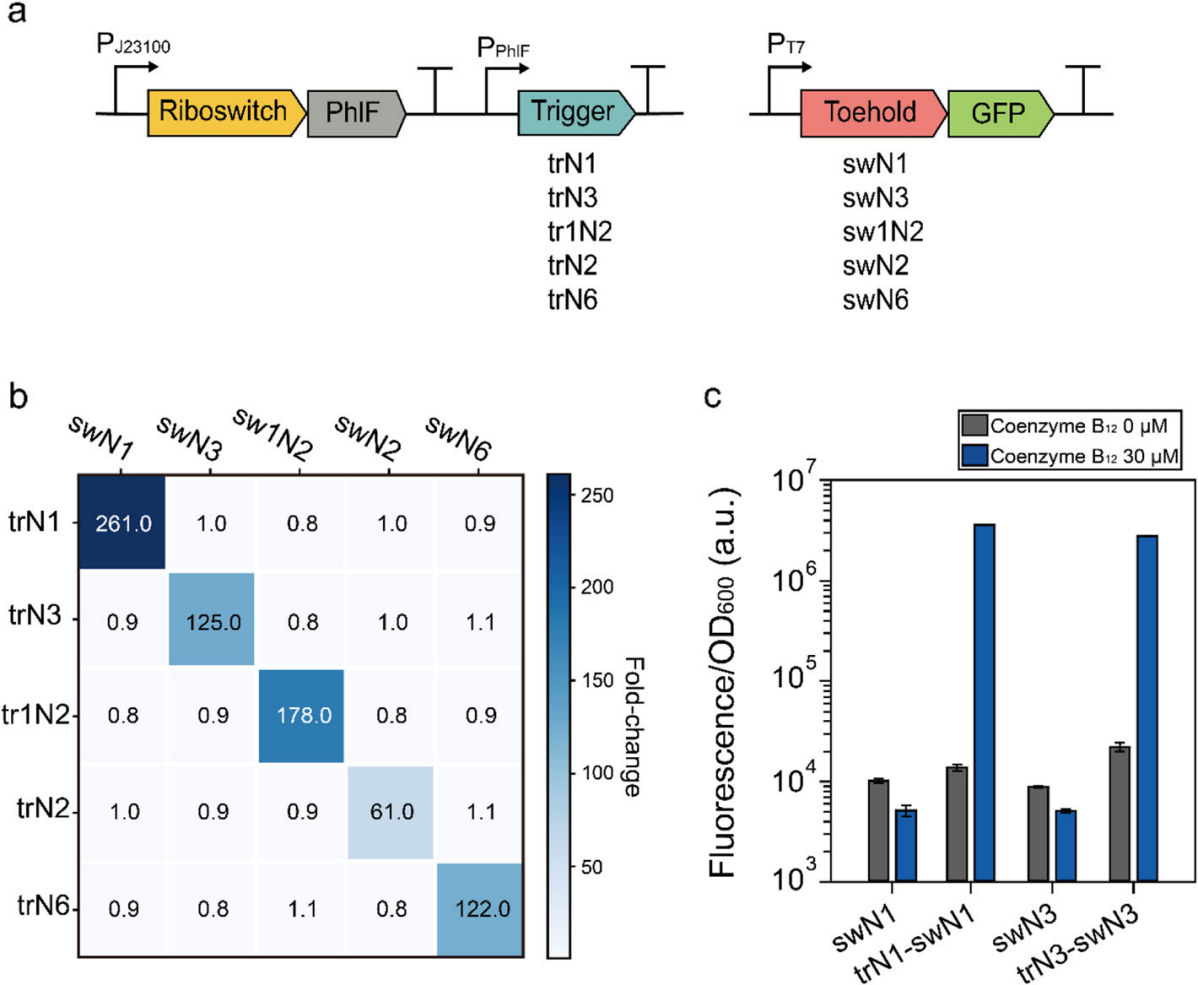
Characterization of riboswitch circuits engineered with toehold switch-based modulators. **a** Scheme of the toehold switch-based modulator circuits. Five AND-computing toehold switches (ACTS_TypeII_N1 [trN1 & swN1], ACTS_TypeII_N3 [trN3 & swN3], ACTS_TypeI_N2 [tr1N2 & sw1N2], ACTS_TypeII_N2 [trN2 & swN2], and ACTS_TypeII_N6 [trN6 & swN6]) were used [23]. The names in the square brackets are used in this study for simplicity. **b** Heatmap for the combinations of trigger-toehold switch pairs with or without coenzyme B_12_ (30 μM). The heatmap was drawn using the average fold-change from biological triplicate experiments, and the raw data are listed in Table S3. **c** Fluorescence measurements for switch-only circuits with or without coenzyme B_12_ (30 μM). The 10 μM IPTG was used for all tests in Figure 3. Error bars indicate standard deviations from biological triplicate measurements

To evaluate the scalability of the toehold switch-based modulator approach, four additional toehold switch pairs (trN3-swN3, tr1N2-sw1N2, trN2-swN2, and trN6-swN6) [23] were integrated with the P100 circuit (Fig. 3a), and the fluorescence intensity was measured. All constructs successfully amplified the signal from the hybrid input part exhibiting distinct fold-changes, ranging from 61 to 261, depending on the toehold switch pairs introduced (Fig. 3b, diagonal).

Next, we tested the leakiness and orthogonality of the toehold switch-based modulators. In the absence of coenzyme B_12_, the circuits with cognate pairs showed almost the same fluorescence intensity as the circuits without the trigger RNAs (swN1 and swN3) (Fig. 3c), and even the negative control (NC) which does not contain any fluorescent protein gene (Table S3), validating the low leakiness of the toehold switch-based modulators. Furthermore, the circuits with non-cognate trigger-toehold switch pairs were not responsive to coenzyme B_12_, and GFP expression was induced only when the cognate trigger and switch pairs were introduced (Fig. 3b). The circuits showed consistent results when the fluorescence was measured at single-cell level using flow cytometry (Figure S3). Together, these results indicate that the operation of the engineered coenzyme B_12_ riboswitch circuit requires specific binding of the trigger RNA and its cognate toehold switch, which is consistent with the strong orthogonality of toehold switches, as demonstrated in other studies [22, 23]. This result indicates that the various sets of toehold switches can be used to construct RNA-based genetic modulators.

**Fig. 4:**
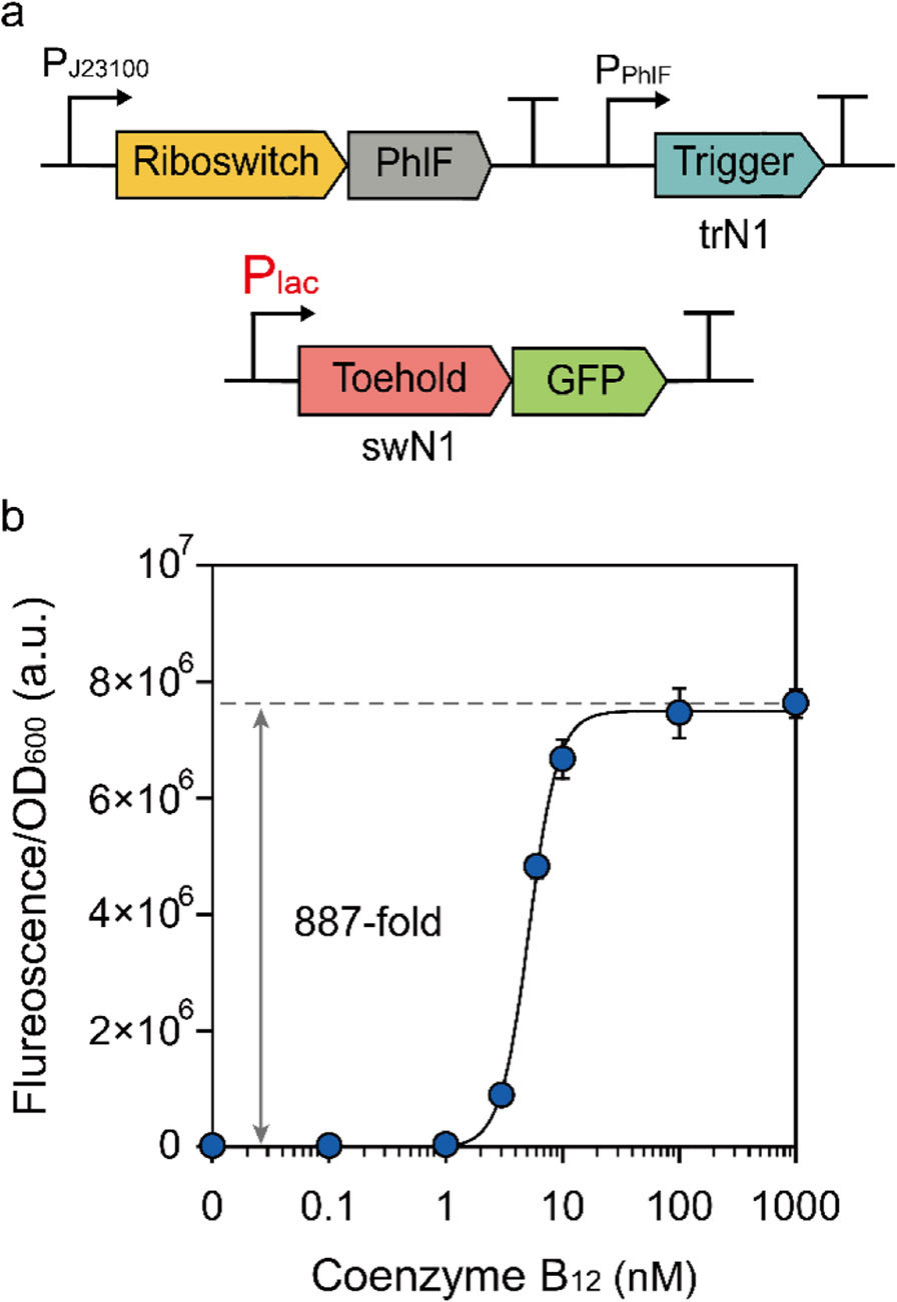
**a** Schematic of the toehold switch-based modulator with the P_lac_ promoter. Toehold switch-based modulator plasmids pACYC-J23100-B12ribo-*phlF*-trN1 and pCOLA-P_lac_-swN1-*gfp* were used for Strain trN1-swN1_L. **b** Dose-response curves of trN1-swN1_L with different concentrations of coenzyme B_12_ (0, 0.1, 1, 3, 6, 10, 100, and 1000 nM) and 100 μM IPTG. Error bars indicate standard deviations from triplicate biological measurements

Finally, we examined the toehold switch-based modulator circuit in a non-RNase-deficient strain to confirm the compatibility of various strains (Figure S4). When tested in *E. coli* BL21 (DE3), both the minimum and maximum output levels were lower than those observed in the RNase-deficient BL21 Star (DE3) because an intact RNase E gene was present in the BL21 (DE3) strain. While the maximum output levels decreased in the BL21 (DE3) strain, the fold-changes of the circuits were increased compared to the BL21 Star (DE3) strain. Then, we evaluated whether the toehold switch-based modulator can operate in the W3110 strain as well. To test toehold switch-based modulators in strains that do not possess T7 RNA polymerase (T7 RNAP) cassette, we changed the promoter of the toehold switch from the T7 promoter to the lac promoter (P_lac_) (Fig. 4a). As a result, the modified circuit showed 887-fold in the W3110 strain (Fig. 4b), suggesting that the toehold switch-based modulator is widely applicable in various strains without genetically encoded T7 RNAP. Interestingly, the operational range – the ligand concentration range in which a biosensor shows distinguishable responses – of the circuit differed dramatically in the BL21 Star (DE3) and W3110 strains. We speculate that this clear difference might be due to the importing systems for coenzyme B_12_ in each strain. The *E. coli* BL21 (DE3), unlike the W3110 strain, has a premature stop codon in the *btuB* gene [28], which plays an essential role in coenzyme B_12_ import [9, 29]. Therefore, the strain BL21 (DE3) and BL21 star (DE3) are considered to have operational ranges different from the strain W3110, because the importing system for coenzyme B_12_ is impaired in those strains.

We used a toehold switch-based modulator to adjust the fold-change in a modular fashion without utilizing prior knowledge of the riboswitch. The fold-change was substantially improved over the previous implementation of the hybrid riboswitch circuit, and the fold-change can be further adjusted by multiple tuning strategies. While we demonstrated the use of high-performance toehold switches and trigger RNA pairs-based modulators in this study, an extended library of more than 100 toehold switches has been reported with more than 20 toehold switch-trigger pairs with a high level of fold-change and orthogonality [22, 23, 30, 31]. Therefore, the use of a toehold switch-based modulator is a highly promising strategy for future efforts to tune the dose-response of genetic circuits to construct more complex genetic programs.

## Conclusion

We demonstrated that a toehold switch-based modulator could tune fold-change of riboswitch-based genetic circuits. The successful incorporation of several pairs of toehold switches and multiple tuning strategies, including adjustments of RNA expression levels, provided evidence for more general applicability of the proposed modulator strategy. In the future, through combined efforts, including mathematical and thermodynamic modeling, the toehold switches may be able to provide modular plug-and-play genetic parts for tuning the riboswitch circuit, expanding the range of applications of synthetic biosensors.

## Materials and methods

### Strains, plasmids, and oligonucleotides

The strains and plasmids used in this study are listed in Table S1, and the oligonucleotides used are listed in Table S2. Sequencing of the constructed plasmids and synthesis of oligonucleotides were performed by Cosmogenetech (Seoul, Korea).

### Bacterial strains and genetic circuit construction

Because the plasmid that regulates the expression of the trigger by coenzyme B_12_ should not contain the T7 promoter, the two T7 promoter sites in pACYCDuet-1 were deleted. The plasmid was PCR-amplified using Q5 High-Fidelity DNA Polymerase (New England Biolabs, Ipswich, MA, USA) with 5’-end phosphorylated primers followed by blunt-end ligation with Quick Ligase (NEB). T7–1-remove-F, R, and T7–2-remove-F and R were used, respectively. Phosphorylation at the 5’-end was performed using T4 Polynucleotide Kinase (Takara, Shiga, Japan).

The coenzyme B_12_ sensing module and trigger moiety of pET-trN1 were inserted into pACYC-dT7 with T7 promoter deletion [23]. The plasmid pB12ribo-J23100-PhlF was used as a template for amplification of the insert using pACYC-gibson-In-F/trN1-R for trN1, where the trigger sequence was included as overhangs in the primers. The vector was amplified using pACYC-dT7 as a template using pACYC-gibson-Ve-R/trN1-F as primers. These amplified fragments were ligated by the Gibson Assembly method using NEBuilder® HiFi DNA Assembly Master Mix (NEB). The resulting coenzyme B_12_ sensing module, pACYC-J23100-B12ribo-*PhlF*-trN1, was co-transformed with pCOLA-swN1-GFP to construct the trN1-swN1 strain (toehold switch ACTS_TypeII_N1 was used) [23].

The pACYC-J23100-B12ribo- *phlF*-trN1 was used as a template for PCR to change the trigger sequence in the coenzyme B_12_ sensing module. DNA fragments with replaced sequences were amplified using the primers Tri-over-trN3-F/R, tr1N2-F/R, trN2-F/R, and trN6-F/R, respectively, and plasmids were constructed by blunt-end ligation. The resulting plasmids, pACYC-J23100-B12ribo-*phlF*-trN3, tr1N2, trN2, and trN6, were co-transformed with pCOLA-swN3, sw1N2, swN2, and swN6-*gfp*, respectively, to construct trN3-swN3, tr1N2-sw1N2, trN2-swN2, and trN6-swN6 strains. The toehold switches used for each strain are ACTS_TypeII_N3, ACTS_TypeI_N2, ACTS_TypeII_N2, and ACTS_TypeII_N6, respectively [23].

The plasmids pACYC-J23119-B12ribo-phlF-trN1 and pACYC-J23101-B12ribo-phlF-trN1 were constructed by blunt-end ligation using the template plasmid pACYC-J23100-B12ribo- *phlF*-trN1 and primers Promoter-change-F/ J23119-R and Promoter-change-F/ J23101-R, respectively. The plasmids pACYC-J23119-B12ribo-phlF-trN1 and pACYC-J23101-B12ribo-phlF-trN1 were co-transformed with pCOLA-swN1-GFP for strain trN1-swN1–119 and trN1-swN1–101, respectively. Additionally, the plasmid pACYC-J23100-B12ribo-*phlF*-deltr was constructed by blunt-end ligation of the PCR product, which was amplified using pACYC-B12ribo-*phlF*-trN1 as a template and del-tr-F/R as primers. For the strain trN1-swN1_L, the two plasmids pACYC-J23100-B12ribo-PhlF-trN1 and pCOLA-P_lac_-swN1-*gfp* were co-transformed to W3110. All genetic circuit systems were tested using *E. coli* BL21 Star (DE3), except for those shown in Fig. 4, for which W3110 and Figure S4 for which *E. coli* BL21 (DE3) was used.

### Fluorescence measurement with coenzyme B_12_

All cultivation experiments were performed using M9 medium containing glucose (4 g/L glucose, 6.78 g/L disodium phosphate (anhydrous), 3 g/L monopotassium phosphate, 0.5 g/L sodium chloride, 1 g/L ammonium chloride, 2 mM magnesium sulfate, 0.1 mM calcium chloride), and appropriate antibiotics (27 mg/L chloramphenicol and 40 mg/L kanamycin). The strains were incubated at 37 °C with shaking at 200 rpm. Single colonies were inoculated into M9 medium, cultured for 24 h, and diluted to a final OD_600_ of 0.05 in fresh M9 medium. Seed cultures in mid-log phase were adjusted to an OD_600_ of 0.05 in fresh M9 medium and incubated for 12 h at 37 °C with shaking at 900 rpm using a 96-Deep-well pate (BIOFIL, Guangzhou, China) and light duty orbital shakers (OHAUS, Seoul, Korea) for Fig. 2. All other tests were done using a test tube at 37 °C with shaking at 200 rpm. Coenzyme B_12_ (Sigma-Aldrich, St. Louis, MO, USA) was added at different concentrations (0, 0.1, 0.3, 1, 3, 10, and 30 μM). For Fig. 4b, coenzyme B_12_ concentrations (0, 0.1, 1, 3, 6, 10, 100, and 1000 nM) were used. Toehold switches connected to GFP reporters were expressed in BL21 Star (DE3) cells, an RNase-deficient strain, or in BL21 (DE3) cells, a non-RNase-deficient strain, with the T7 RNA polymerase induced by adding IPTG at 10 μM unless stated otherwise.

The fluorescence and OD_600_ of the cells were measured using a VICTOR^3^ 1420 Multilabel Counter (PerkinElmer, Waltham, MA, USA). First, cell pellets were washed with phosphate-buffered saline (PBS) and resuspended in PBS. Fluorescence was then measured using a 485-nm excitation filter and 535-nm emission filter with a 0.1^-s^ measurement time, and the OD_600_ was determined using a 600-nm filter, both with a 0.1^-s^ measurement time. The OD_600_ and fluorescence values were corrected by subtracting the values measured for PBS. The autofluorescence of the cells was not subtracted from the fluorescence value.

The CytoFLEX_Plate Loader (Beckman Coulter, Brea, CA, USA) was used to measure the fluorescence of 20,000 cells per sample. A 488-nm laser was used to excite the 525/40 bandpass filter to measure FITC-A (Fluorescein isothiocyanate-area), and the gate was selected by FSC-A (Forward scattered light-area) and SSC-A (Side scatter-area) to filter out the other impurities. Finally, the histogram (Figure S3) was drawn with the cell count and FITC-A value measured in the selected gate.

### Fitting of dose-response curves and calculation of EC_50_

SigmaPlot software (Systat Software, Inc., San Jose, CA, USA) was used to fit the dose-response curve. Data were fitted using a nonlinear regression – dynamic fitting program, and an equation of ligand binding and sigmoidal dose-response was selected. The EC_50_ value was calculated using the fitting results, and the following logistic equation was used: Fluorescence = Min. + (Max. - Min.)/(1 + 10^(log(EC50)-log(coenzyme B12)) × (Hill coefficient)^).

## Supplementary Information

**Additional file 1: Table S1.** Strains and plasmids used in this study. **Table S2.** Oligonucleotides used in this study. **Table S3.** Measured OD_600_, GFP fluorescence, and specific fluorescence. **Figure S1.** Overall schematics of the toehold switch. Switch RNA is hiding the RBS (ribosome binding site) in the hairpin structure. When trigger RNA binds complementarily to the toehold domain within switch RNA [A], the switch RNA stem starts to unwind, exposing RBS and the start codon to activate translation of the reporter gene. **Figure S2.** (a) Dose-response curves for trN1-swN1with various IPTG (1, 10, 100, 1000 μM) and coenzyme B_12_ (0, 0.1, 0.3, 1, 3, 10, 30 μM) concentrations. (b) Dose-response curves for modified trN1-swN1 strain in which the promoter for the riboswitch was changed to J23119 with various IPTG (1, 10, 100, 1000 μM) and coenzyme B_12_ (0, 0.1, 0.3, 1, 3, 10, 30 μM) concentrations. (c) EC_50_ values for (a). (d) EC_50_ Values for (b). Error bars indicate standard deviations from triplicate measurements. **Figure S3.** Histograms of the toehold switch-based modulators measured by flow cytometry. The fold-changes of average fluorescence in the presence and absence of coenzyme B_12_ are plotted in Fig. 3b. The strain NC (negative control) refers to BL21 star (DE3) without toehold switch-based modulator. **Figure S4.** Performance of toehold switch-based modulator in *E. coli* BL21(DE3). (a) Fluorescence measurements for BL21-trN1-swN1 and BL21-trN3-swN3 with and without coenzyme B_12_ (30 μM). (b) Fold-change of GFP reporters for BL21-trN1-swN1 and BL21-trN3-swN3. Error bars indicate standard deviations from triplicate measurements.

## Data Availability

All data generated or analyzed during this study are included in this published article and its additional information files.

## Abbreviations

P_J23xxx_: Anderson constitutive promoter series
P_PhlF_: PhlF transcriptional repressor cognate promoter
P_T7_: T7 promoter
TX: Transcription
TL: Translation
GFP: Green fluorescent protein
RBS: Ribosomal binding site
IPTG: Isopropyl β-D-1-thiogalactopyranoside
EC_50_: Half maximal effective concentration
T7 RNAP: T7 RNA polymerase
P_lac_: Lac promoter
OD_600_: Optical density at 600 nm
PBS: Phosphate-buffered saline
FITC-A: Fluorescein isothiocyanate-area
FSC-A: Forward scattered light-area
SSC-A: Side scatter-area
