# Supplementary material for: Signal amplification and optimization of riboswitch-based hybrid inputs by modular and titratable toehold switches
Source: J Biol Eng. 2021 Mar 19;15:11. doi: 10.1186/s13036-021-00261-w (PMC7977183; doi:10.1186/s13036-021-00261-w)
Supplement: Supplementary file 1 — Additional file 1: Table S1. Strains and plasmids used in this study. Table S2. Oligonucleotides used in this study. Table S3. Measured OD600, GFP fluorescence, and specific fluorescence. Figure S1. Overall schematics of the toehold switch. Switch RNA is hiding the RBS (ribosome binding site) in the hairpin structure. When trigger RNA binds complementarily to the toehold domain within switch RNA [A], the switch RNA stem starts to unwind, exposing RBS and the start codon to activate translation of the reporter gene. Figure S2. (a) Dose-response curves for trN1-swN1with various IPTG (1, 10, 100, 1000 μM) and coenzyme B12 (0, 0.1, 0.3, 1, 3, 10, 30 μM) concentrations. (b) Dose-response curves for modified trN1-swN1 strain in which the promoter for the riboswitch was changed to J23119 with various IPTG (1, 10, 100, 1000 μM) and coenzyme B12 (0, 0.1, 0.3, 1, 3, 10, 30 μM) concentrations. (c) EC50 values for (a). (d) EC50 Values for (b). Error bars indicate standard deviations from triplicate measurements. Figure S3. Histograms of the toehold switch-based modulators measured by flow cytometry. The fold-changes of average fluorescence in the presence and absence of coenzyme B12 are plotted in Fig. 3b. The strain NC (negative control) refers to BL21 star (DE3) without toehold switch-based modulator. Figure S4. Performance of toehold switch-based modulator in E. coli BL21(DE3). (a) Fluorescence measurements for BL21-trN1-swN1 and BL21-trN3-swN3 with and without coenzyme B12 (30 μM). (b) Fold-change of GFP reporters for BL21-trN1-swN1 and BL21-trN3-swN3. Error bars indicate standard deviations from triplicate measurements. [file 13036_2021_261_MOESM1_ESM.docx]

**Supporting Information for:**

**Signal amplification and optimization of riboswitch-based hybrid inputs by modular and titratable toehold switches**

Yunhee Hwang^a, 1^, Seong Gyeong Kim^a, 1^, Sungho Jang^b, c, 1^, Jongmin Kim^d, *^,
Gyoo Yeol Jung^a, e, **^

^a^ Department of Chemical Engineering, Pohang University of Science and Technology, 77 Cheongam-ro, Nam-gu, Pohang, Gyeongbuk 37673, Korea

^b^ Department of Bioengineering and Nano-Bioengineering, Incheon National University, 119 Academy-ro, Yeonsu-gu, Incheon 22012, Korea

^c^ Division of Bioengineering, College of Life Sciences and Bioengineering, Incheon National University, 119 Academy-ro, Yeonsu-gu, Incheon 22012, Korea

^d^ Integrative Biosciences and Biotechnology, Pohang University of Science and Technology, 77 Cheongam-ro, Nam-gu, Pohang, Gyeongbuk 37673, Korea

^e^ School of Interdisciplinary Bioscience and Bioengineering, Pohang University of Science and Technology, 77 Cheongam-ro, Nam-gu, Pohang, Gyeongbuk 37673, Korea

^*^ Corresponding author: Jongmin Kim Mailing address: Division of Integrative Biosciences and Biotechnology, Pohang University of Science and Technology, 77 Cheongam-ro, Nam-gu, Pohang, Gyeongbuk 37673, Korea

^**^ Corresponding author: Gyoo Yeol Jung Mailing address: Department of Chemical Engineering, Pohang University of Science and Technology, 77 Cheongam-ro, Nam-gu, Pohang, Gyeongbuk 37673, Korea

E-mail address: [jongmin.kim@postech.ac.kr](mailto:jongmin.kim@postech.ac.kr) (J. Kim); [gyjung@postech.ac.kr](mailto:gyjung@postech.ac.kr) (G. Y. Jung)

Telephone: +82-54-279-2322 (J. Kim); +82-54-279-2391 (G. Y. Jung)

^1^ These authors contributed equally to this work.

**Contents:**

Table S1, S2, and S3

Figure S1, S2, S3 and S4

**Table S1.** Strains and plasmids used in this study

| Name | Relevant characteristics | Source |
| --- | --- | --- |
| **Strains** |  |  |
| Mach-T1^R^ | F^-^ φ80(*lac*Z)ΔM15 Δ*lac*X74 *hsd*R(r_K_-mK+) Δ*rec*A1398 *end*A1 *ton*A | Invitrogen |
| BL21(DE3) | F^–^ *ompT* *gal* *dcm* *hsdS_B_*(*r_B_*^–^*m_B_*^–^) (DE3) | Invitrogen |
| BL21 Star (DE3) | F^–^ *ompT* *gal* *dcm* *hsdS_B_*(*r_B_*^–^*m_B_*^–^) *rne*131 (DE3) | Invitrogen |
| W3110 | F^-^ λ^-^ rph-1 IN(*rrnD*, *rrnE*) | ATCC27325 |
| P100 | W3110/pB12ribo-J23100-PhlF | Jang, S et al., 2018 |
| trN1-swN1 | BL21 Star (DE3)/pACYC-J23100-B12ribo-*phlF*-trN1/ pCOLA-swN1-*gfp* | This study |
| trN3-swN3 | BL21 Star (DE3)/pACYC-J23100-B12ribo- *phlF* -trN3/ pCOLA-swN3-*gfp* | This study |
| tr1N2-sw1N2 | BL21 Star (DE3)/pACYC-J23100-B12ribo- *phlF* -tr1N2/pCOLA-sw1N2-*gfp* | This study |
| trN2-swN2 | BL21 Star (DE3)/pACYC-J23100-B12ribo- *phlF* -trN2/pCOLA-swN2-*gfp* | This study |
| trN6-swN6 | BL21 Star (DE3)/pACYC-J23100-B12ribo- *phlF* -trN6/pCOLA-swN6-*gfp* | This study |
| trN1-swN1-119 | BL21 Star (DE3)/pACYC-J23119-B12ribo-*phlF*-trN1/ pCOLA-swN1-*gfp* | This study |
| trN1-swN1-101 | BL21 Star (DE3)/pACYC-J23101-B12ribo-*phlF*-trN1/ pCOLA-swN1-*gfp* | This study |
| swN1 | BL21 Star (DE3)/pACYC-J23100-B12ribo- *phlF* -deltr/ pCOLA-swN1-*gfp* | This study |
| swN3 | BL21 Star (DE3)/pACYC-J23100-B12ribo- *phlF* -deltr/ pCOLA-swN3-*gfp* | This study |
| trN1-swN3 | BL21 Star (DE3)/pACYC-J23100-B12ribo- *phlF* -trN1/ pCOLA-swN3-*gfp* | This study |
| trN1-sw1N2 | BL21 Star (DE3)/pACYC-J23100-B12ribo- *phlF* -trN1/ pCOLA-sw1N2-*gfp* | This study |
| trN1-swN2 | BL21 Star (DE3)/pACYC-J23100-B12ribo- *phlF* -trN1/ pCOLA-swN2-*gfp* | This study |
| trN1-swN6 | BL21 Star (DE3)/pACYC-J23100-B12ribo- *phlF* -trN1/ pCOLA-swN6-*gfp* | This study |
| trN3-swN1 | BL21 Star (DE3)/pACYC-J23100-B12ribo- *phlF* -trN3/ pCOLA-swN1*-gfp* | This study |
| trN3-sw1N2 | BL21 Star (DE3)/pACYC-J23100-B12ribo- *phlF* -trN3/ pCOLA-sw1N2*-gfp* | This study |
| trN3-swN2 | BL21 Star (DE3)/pACYC-J23100-B12ribo- *phlF* -trN3/ pCOLA-swN2*-gfp* | This study |
| trN3-swN6 | BL21 Star (DE3)/pACYC-J23100-B12ribo- *phlF* -trN3/ pCOLA-swN6*-gfp* | This study |
| tr1N2-swN1 | BL21 Star (DE3)/pACYC-J23100-B12ribo- *phlF* -tr1N2/ pCOLA-swN1*-gfp* | This study |
| tr1N2-swN3 | BL21 Star (DE3)/pACYC-J23100-B12ribo- *phlF* -tr1N2/ pCOLA-swN3*-gfp* | This study |
| tr1N2-swN2 | BL21 Star (DE3)/pACYC-J23100-B12ribo- *phlF* -tr1N2/ pCOLA-swN2*-gfp* | This study |
| tr1N2-swN6 | BL21 Star (DE3)/pACYC-J23100-B12ribo- *phlF* -tr1N2/ pCOLA-swN6*-gfp* | This study |
| trN2-swN1 | BL21 Star (DE3)/pACYC-J23100-B12ribo- *phlF* -trN2/ pCOLA-swN1*-gfp* | This study |
| trN2-swN3 | BL21 Star (DE3)/pACYC-J23100-B12ribo- *phlF* -trN2/ pCOLA-swN3*-gfp* | This study |
| trN2-sw1N2 | BL21 Star (DE3)/pACYC-J23100-B12ribo- *phlF* -trN2/ pCOLA-sw1N2*-gfp* | This study |
| trN2-swN6 | BL21 Star (DE3)/pACYC-J23100-B12ribo- *phlF* -trN2/ pCOLA-swN6*-gfp* | This study |
| trN1-swN1_L | W3110/pACYC-J23100-B12ribo-*phlF*-trN1/ pCOLA-P_lac_-swN1-*gfp* | This study |
| BL-trN1-swN1 | BL21 (DE3)/pACYC-J23100-B12ribo-*phlF*-trN1/ pCOLA-swN1-*gfp* | This study |
| BL-trN3-swN3 | BL21 (DE3)/pACYC-J23100-B12ribo- *phlF* -trN3/ pCOLA-swN3-*gfp* | This study |
|  |  |  |
| **Plasmids** |  |  |
| pACYCDuet | Expression vector, Cm^R^, p15A ori | Novagen |
| pCOLADuet | Expression vector, Km­^R^, ColA ori | Novagen |
| pB12ribo-J23100-PhlF | pACYCDuet-P_BBa_J23100­_-*cbiA_rib-phlF-*P_PhlF_*-tetAsgfp* | Jang, S. et al., 2018 |
| pET-trN1 | pET-P_T7_-trigger_TypeII_N1 | Green, A.A. et al., 2017 |
| pCOLA-swN1-*gfp* | pCOLA-P_T7_-switch_TypeII_N1-*gfpmut3b-asv* | Green, A.A. et al., 2017 |
| pACYC-dT7 | pACYCDuet-ΔT7 | This study |
| pACYC-J2300-B12ribo-*phlF*-trN1 | pACYCDuet-P_BBa_J23100­_-*cbiA_rib-phlF-*P_PhlF_*-*trigger_TypeII_N1 | This study |
| pCOLA-swN3-*gfp* | pCOLA-P_T7_-switch_TypeII_N3-*gfpmut3b-asv* | Green, A.A. et al., 2017 |
| pCOLA-sw1N2-*gfp* | pCOLA-P_T7_-switch_TypeI_N2-*gfpmut3b-asv* | Green, A.A. et al., 2017 |
| pCOLA-swN2-*gfp* | pCOLA-P_T7_-switch_TypeII_N2-*gfpmut3b-asv* | Green, A.A. et al., 2017 |
| pCOLA-swN6-*gfp* | pCOLA-P_T7_-switch_TypeII_N6-*gfpmut3b-asv* | Green, A.A. et al., 2017 |
| pACYC-J23100-B12ribo-*phlF*-trN3 | pACYCDuet-P_BBa_J23100­_-*cbiA_rib-phlF-*P_PhlF_*-*trigger_TypeII_N3 | This study |
| pACYC-J23100-B12ribo-*phlF*-tr1N2 | pACYCDuet-P_BBa_J23100­_-*cbiA_rib-phlF-*P_PhlF_*-*trigger_TypeI_N2 | This study |
| pACYC-J23100-B12ribo-*phlF*-trN2 | pACYCDuet-P_BBa_J23100­_-*cbiA_rib-phlF-*P_PhlF_*-*trigger_TypeII_N2 | This study |
| pACYC-J23100-B12ribo-*phlF*-trN6 | pACYCDuet-P_BBa_J23100­_-*cbiA_rib-phlF-*P_PhlF_*-*trigger_TypeII_N6 | This study |
| pACYC-J23119-B12ribo-*phlF*-trN1 | pACYCDuet-P_BBa_J23119­_-*cbiA_rib-phlF-*P_PhlF_*-*trigger_TypeII_N1 | This study |
| pACYC-J23101-B12ribo-*phlF*-trN1 | pACYCDuet-P_BBa_J23101­_-*cbiA_rib-phlF-*P_PhlF_*-*trigger_TypeII_N1 | This study |
| pACYC-J23100-B12ribo-*phlF*-deltr | pACYCDuet-P_BBa_J23100­_-*cbiA_rib-phlF* | This study |
| pCOLA-P_lac_-swN1-*gfp* | pCOLA-P_llac-O_-switch_TypeII_N1-*gfpmut3b-asv* | Green, A.A. et al., 2017 |

Sequences written in relevant characteristics triggerTypeI_N2 and switch_TypeI_N2, trigger_TypeII_N1 & switch_TypeII_N1, trigger_TypeII_N2 & switch_TypeII_N2, trigger_TypeII_N3 & switch_TypeII_N3, trigger_TypeII_N6, and switch_TypeII_N6 are ACTS_TypeI_N2, ACTS_TypeII_N1, ACTS_TypeII_N2, ACTS_TypeII_N3, ACTS_TypeII_N6 used at Green, A.A. et al., 2017, respectively.

**Table S2.** Oligonucleotides used in this study

| Name | Sequence (5′-3′)^a^ |
| --- | --- |
| T7-1-remove-F | CCTGTAGAAATAATTTTGTTTAACTTTAATAAGG |
| T7-1-remove-R | ATTTCCTAATGCAGGAGTCGC |
| T7-2-remove-F | CCATCTTAGTATATTAGTTAAGTATAAGAAGGAG |
| T7-2-remove-R | ATTTCGATTATGCGGCC |
| pACYC-gibson-In-F | TCACCACAGCCAGGATCCGAATTCGAGCTCTTGACGGCTAGCTCAGTCCTAG |
| pACYC-gibson-In-trN1-R | TGAAGGTAGGTATGTTCGTCTCGATCAGATTAGTGATCGAGCACCTTAACGATACGGTACGTTTCG |
| pACYC-gibson-Ve-trN1-F | ATCTGATCGAGACGAACATACCTACCTTCATTATCTTACTTGTCTAGCATAACCCCTTGGGGC |
| pACYC-gibson-Ve-R | GAGCTCGAATTCGGATCCTG |
| Tri-over-trN3-F | TACTAAACTTCTATCATATTCAATCACCTAGCATAACCCCTTGGGGCCTC |
| Tri-over-trN3-R | GTGTTATACACATGATTCTATGTGTATCACCTTAACGATACGGTACGTTTCGTATC |
| Tri-over-tr1N2-F | AATAGAATTGAACATAGATAAGCTAGCATAACCCCTTGGGGCCTC |
| Tri-over-tr1N2-R | CGTCGTCTCTACACCGGTCTCTGCAAAGACCGGTCACCTTAACGATACGGTACGTTTCGTATC |
| Tri-over-trN2-F | TAGAACGTATTACACTCATAAGATACTAGCATAACCCCTTGGGGCCTC |
| Tri-over-trN2-R | TGTCCAGAGTCTTCATTATCTTGAAGACTCACCTTAACGATACGGTACGTTTCGTATC |
| Tri-over-trN6-F | CACTAACTACTTTCGCATGTCTCATCTTCTAGCATAACCCCTTGGGGCCTC |
| Tri-over-trN6-R | TTCGTAGACTCAGGCCAGTGAGTCTACACCTTAACGATACGGTACGTTTCGTATC |
| Promoter-change-F | ACGTAAACCAACAGGTTTGCCACA |
| J23119-R | GCTAGCATTATACCTAGGACTGAGCTAGCTGTCAAAGCTTGTCGACCTGCAGGCGC |
| J23101-R | GCTAGCATAATACCTAGGACTGAGCTAGCTGTAAAAGCTTGTCGAGCTCGAATTCGGATCCTG |
| del-tr-F | GGTCTTGAGGGGTTTTTTGC |
| del-tr-R | GAGAGCGTTCACCGACAAAC |
|  |  |
|  |  |

^a^ Underlined letters indicate the homology sequence used for Gibson Assembly

**Table S3.** Measured OD_600_, GFP fluorescence, and specific fluorescence

| Strains &  IPTG (µM) | Coenzyme B_12_ | OD_600_^a^ | Fluorescence^b^ | Specific fluorescence^c^ |
| --- | --- | --- | --- | --- |
| **Figure 2** |  |  |  |  |
| **IPTG (1 µM)** |  |  |  |  |
| trN1-swN1  _J23101 | 0 µM | 0.63±0.007 | 631816±20161 | 1008278±28426 |
|  | 30 µM | 0.59±0.007 | 491450±6769 | 837740±5189 |
| trN1-swN1 | 0 µM | 0.67±0.01 | 9121±511 | 13549±793 |
|  | 30 µM | 0.63±0.003 | 403388±8172 | 635994±15238 |
| trN1-swN1  _J23119 | 0 µM | 0.70±0.053 | 12507±1736 | 13549±1284 |
|  | 30 µM | 0.67±0.003 | 535833±14625 | 635994±18786 |
| **IPTG (10 µM)** |  |  |  |  |
| trN1-swN1  _J23101 | 0 µM | 0.60±0.014 | 2580639±58045 | 4285960±59846 |
|  | 30 µM | 0.58±0.013 | 2624362±48959 | 4517104±44914 |
| trN1-swN1 | 0 µM | 0.67±0.009 | 9242±709 | 13821±974 |
|  | 30 µM | 0.62±0.011 | 2255066±21121 | 3611364±43015 |
| trN1-swN1  _J23119 | 0 µM | 0.67±0.011 | 11654±3597 | 17354±834 |
|  | 30 µM | 0.66±0.007 | 2674749±101416 | 4068100±95424 |
| **IPTG (100 µM)** |  |  |  |  |
| trN1-swN1  _J23101 | 0 µM | 0.61±0.046 | 3600909±1009267 | 6007585±2015188 |
|  | 30 µM | 0.55±0.009 | 4026236±47478 | 7301920±81357 |
| trN1-swN1 | 0 µM | 0.65±0.001 | 71898±455 | 110787±755 |
|  | 30 µM | 0.62±0.023 | 4125284±138279 | 6640035±74300 |
| trN1-swN1  _J23119 | 0 µM | 0.64±0.013 | 234058±3597 | 363631±2582 |
|  | 30 µM | 0.65±0.001 | 5363885±101416 | 8203969±150663 |
| **IPTG (1000 µM)** |  |  |  |  |
| trN1-swN1  _J23101 | 0 µM | 0.55±0.003 | 2388461±78544 | 4333045±122177 |
|  | 30 µM | 0.53±0.009 | 2381022±44900 | 4501911±38373 |
| trN1-swN1 | 0 µM | 0.64±0.012 | 47062±1124 | 73919±3039 |
|  | 30 µM | 0.61±0.011 | 1860385±26658 | 3069353±48498 |
| trN1-swN1  _J23119 | 0 µM | 0.64±0.013 | 241802±8813 | 374844±8552 |
|  | 30 µM | 0.63±0.004 | 2643576±12685 | 4228369±29712 |
|  |  |  |  |  |
| **Figure 3b** |  |  |  |  |
| **IPTG (10 µM)** |  |  |  |  |
| trN1-swN1 | 0 µM | 0.67±0.009 | 9242±709 | 13821±974 |
|  | 30 µM | 0.62±0.011 | 2255066±21121 | 3611364±43015 |
| trN1-swN3 | 0 µM | 0.55±0.039 | 8692±1749 | 16134±4246 |
|  | 30 µM | 0.55±0.014 | 8214±735 | 15001±1625 |
| trN1-sw1N2 | 0 µM | 0.54±0.003 | 8983±1278 | 16597±2435 |
|  | 30 µM | 0.53±0.004 | 6880±639 | 12983±1198 |
| trN1-swN2 | 0 µM | 0.58±0.019 | 8555±767 | 14872±1770 |
|  | 30 µM | 0.54±0.011 | 8230±284 | 15158±334 |
| trN1-swN6 | 0 µM | 0.60±0.006 | 8130±450 | 13501±858 |
|  | 30 µM | 0.60±0.018 | 7395±638 | 12426±1051 |
| trN3-swN1 | 0 µM | 0.58±0.027 | 8419±1176 | 14577±2568 |
|  | 30 µM | 0.59±0.009 | 8592±489 | 14535±1009 |
| trN3-swN3 | 0 µM | 0.56±0.013 | 12566±991 | 22299±2209 |
|  | 30 µM | 0.62±0.009 | 1726066±24481 | 2798072±12000 |
| trN3-sw1N2 | 0 µM | 0.58±0.024 | 8741±1422 | 15042±2617 |
|  | 30 µM | 0.61±0.01 | 8565±529 | 14128±649 |
| trN3-swN2 | 0 µM | 0.55±0.027 | 7962±832 | 14475±2136 |
|  | 30 µM | 0.58±0.009 | 7249±314 | 12424±632 |
| trN3-swN6 | 0 µM | 0.61±0.016 | 8629±189 | 14115±668 |
|  | 30 µM | 0.62±0.014 | 7065±629 | 11358±1216 |
| tr1N2-swN1 | 0 µM | 0.56±0.018 | 8435±575 | 15201±1345 |
|  | 30 µM | 0.62±0.012 | 7239±416 | 11738±875 |
| tr1N2-swN3 | 0 µM | 0.54±0.015 | 8929±393 | 16573±1000 |
|  | 30 µM | 0.59±0.017 | 7443±452 | 12672±1101 |
| tr1N2-sw1N2 | 0 µM | 0.56±0.029 | 8248±482 | 14725±1650 |
|  | 30 µM | 0.62±0.015 | 1625251±96805 | 2619292±214071 |
| tr1N2-swN2 | 0 µM | 0.59±0.009 | 9008±1177 | 15264±2146 |
|  | 30 µM | 0.61±0.011 | 8143±295 | 13423±409 |
| tr1N2-swN6 | 0 µM | 0.62±0.029 | 7353±657 | 11966±840 |
|  | 30 µM | 0.600.004 | 7662±456 | 12840±847 |
| trN2-swN1 | 0 µM | 0.60±0.006 | 8417±1071 | 14016±1917 |
|  | 30 µM | 0.66±0.032 | 8915±1111 | 13501±1654 |
| trN2-swN3 | 0 µM | 0.57±0.013 | 7599±1327 | 13391±2641 |
|  | 30 µM | 0.60±0.003 | 7984±574 | 13393±1005 |
| trN2-sw1N2 | 0 µM | 0.59±0.034 | 8428±886 | 14465±2254 |
|  | 30 µM | 0.64±0.007 | 7906±1411 | 12328±2328 |
| trN2-swN2 | 0 µM | 0.54±0.013 | 8051±1266 | 14994±2677 |
|  | 30 µM | 0.58±0.013 | 529603±25217 | 916942±23504 |
| trN2-swN6 | 0 µM | 0.57±0.002 | 8122±388 | 14309±716 |
|  | 30 µM | 0.61±0.014 | 6461±497 | 10702±1081 |
| trN6-swN1 | 0 µM | 0.55±0.014 | 8906±1545 | 16311±3269 |
|  | 30 µM | 0.53±0.005 | 7752±155 | 14577±332 |
| trN6-swN3 | 0 µM | 0.54±0.027 | 7975±138 | 14681±957 |
|  | 30 µM | 0.52±0.008 | 8506±1133 | 16457±2411 |
| trN6-sw1N2 | 0 µM | 0.57±0.012 | 8412±277 | 14741±606 |
|  | 30 µM | 0.55±0.016 | 7011±564 | 12723±1318 |
| trN6-swN2 | 0 µM | 0.58±0.01 | 8272±560 | 14221±934 |
|  | 30 µM | 0.54±0.008 | 8328±587 | 15327±1303 |
| trN6-swN6 | 0 µM | 0.58±0.021 | 8957±1044 | 15383±2219 |
|  | 30 µM | 0.57±0.035 | 1073126±67263 | 1875002±46627 |
| NC^d^ | 0 µM | 0.55±0.007 | 7023±945 | 12693±1647 |
|  | 30 µM | 0.6±0.025 | 6657±876 | 11073±1648 |
|  |  |  |  |  |
| **Figure 3c** |  |  |  |  |
| **IPTG (10 µM)** |  |  |  |  |
| swN1 | 0 µM | 0.69±0.014 | 7190±539 | 10220±585 |
|  | 30 µM | 0.7±0.03 | 3494±292 | 5154±656 |
| trN1-swN1 | 0 µM | 0.67±0.009 | 9242±709 | 13821±974 |
|  | 30 µM | 0.62±0.011 | 2255066±21121 | 3611364±43015 |
| swN3 | 0 µM | 0.68±0.036 | 6204±226 | 8913±165 |
|  | 30 µM | 0.70±0.034 | 3615±293 | 5119±200 |
| trN3-swN3 | 0 µM | 0.56±0.013 | 12566±991 | 22299±2209 |
|  | 30 µM | 0.62±0.009 | 1726066±24481 | 2798072±12000 |
|  |  |  |  |  |
| **Figure 4** |  |  |  |  |
| **IPTG (10 µM)** |  |  |  |  |
| trN1-swN1_L | 0 µM | 0.5±0.012 | 4285±171 | 8595 ± 152 |
|  | 1 µM | 0.49±0.016 | 3728327±199363 | 7629576 ± 247609 |

^a, b^ The average value was calculated by measuring OD_600_ and fluorescence of triplicate samples with VICTOR^3^, respectively. The OD_600_ and fluorescence values of PBS were subtracted.

^c^ The specific fluorescence was calculated with the following formula. $\frac{Fluorescence\left[ sample \right]-Fluorescence\left[ PBS \right]}{{OD}_{600}\left[ Sample \right]-{OD}_{600}[PBS]}$

The final specific fluorescence was calculated as the average value of specific fluorescence intensities of the triplicate samples.

^d^ NC (Negative control) refers to BL21 star (DE3) without toehold switch-based modulator.


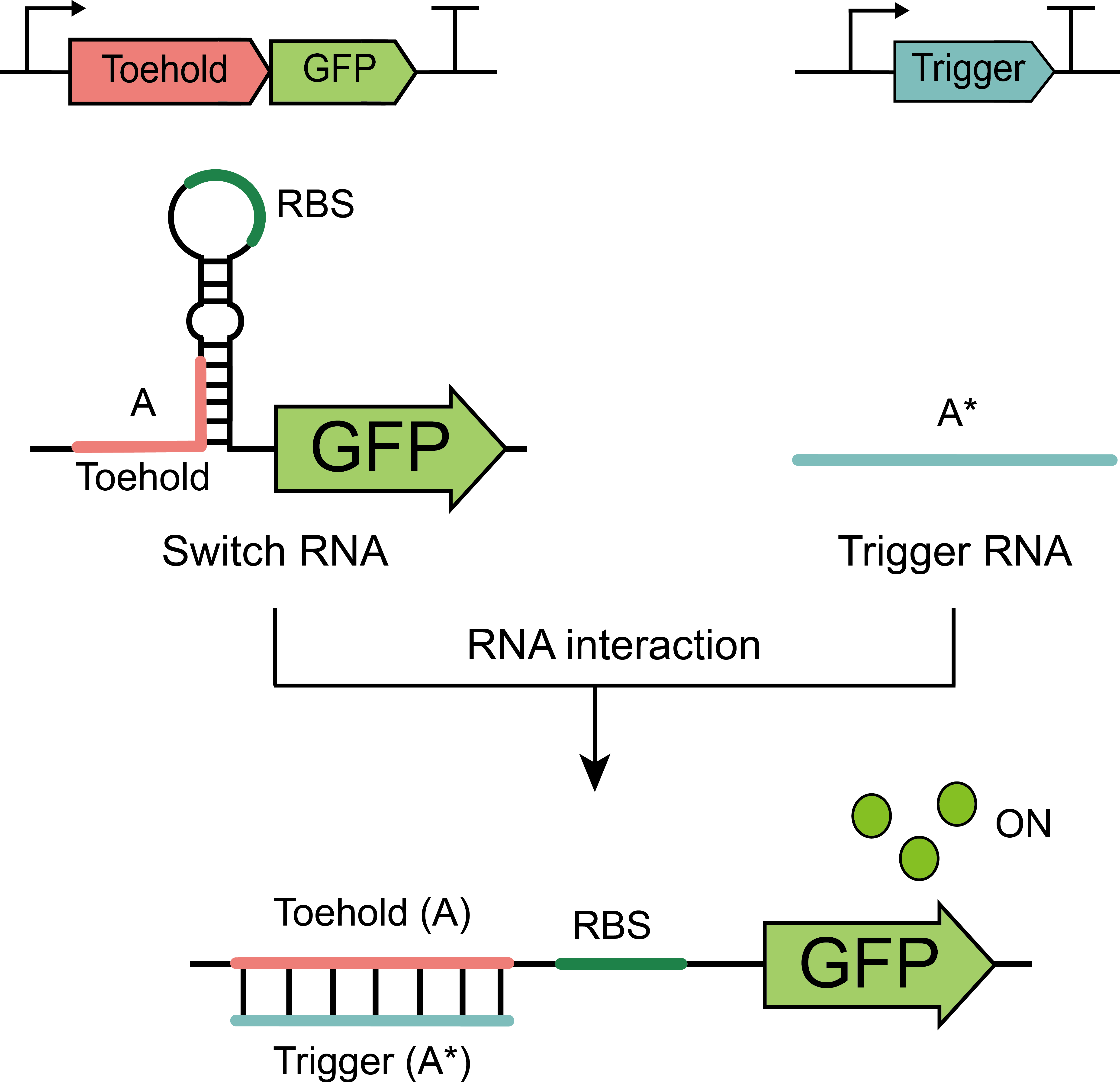


**Figure S1.** Overall schematics of the toehold switch. Switch RNA is hiding the RBS (ribosome binding site) in the hairpin structure. When trigger RNA binds complementarily to the toehold domain within switch RNA [A], the switch RNA stem starts to unwind, exposing RBS and the start codon to activate translation of the reporter gene.





**Figure S2.** (a) Dose-response curves for trN1-swN1with various IPTG (1, 10, 100, 1000 μM) and coenzyme B_12_ (0, 0.1, 0.3, 1, 3, 10, 30 μM) concentrations. (b) Dose-response curves for modified trN1-swN1 strain in which the promoter for the riboswitch was changed to J23119 with various IPTG (1, 10, 100, 1000 μM) and coenzyme B_12_ (0, 0.1, 0.3, 1, 3, 10, 30 μM) concentrations. (c) EC_50_ values for (a). (d) EC_50_ Values for (b). Error bars indicate standard deviations from triplicate measurements.


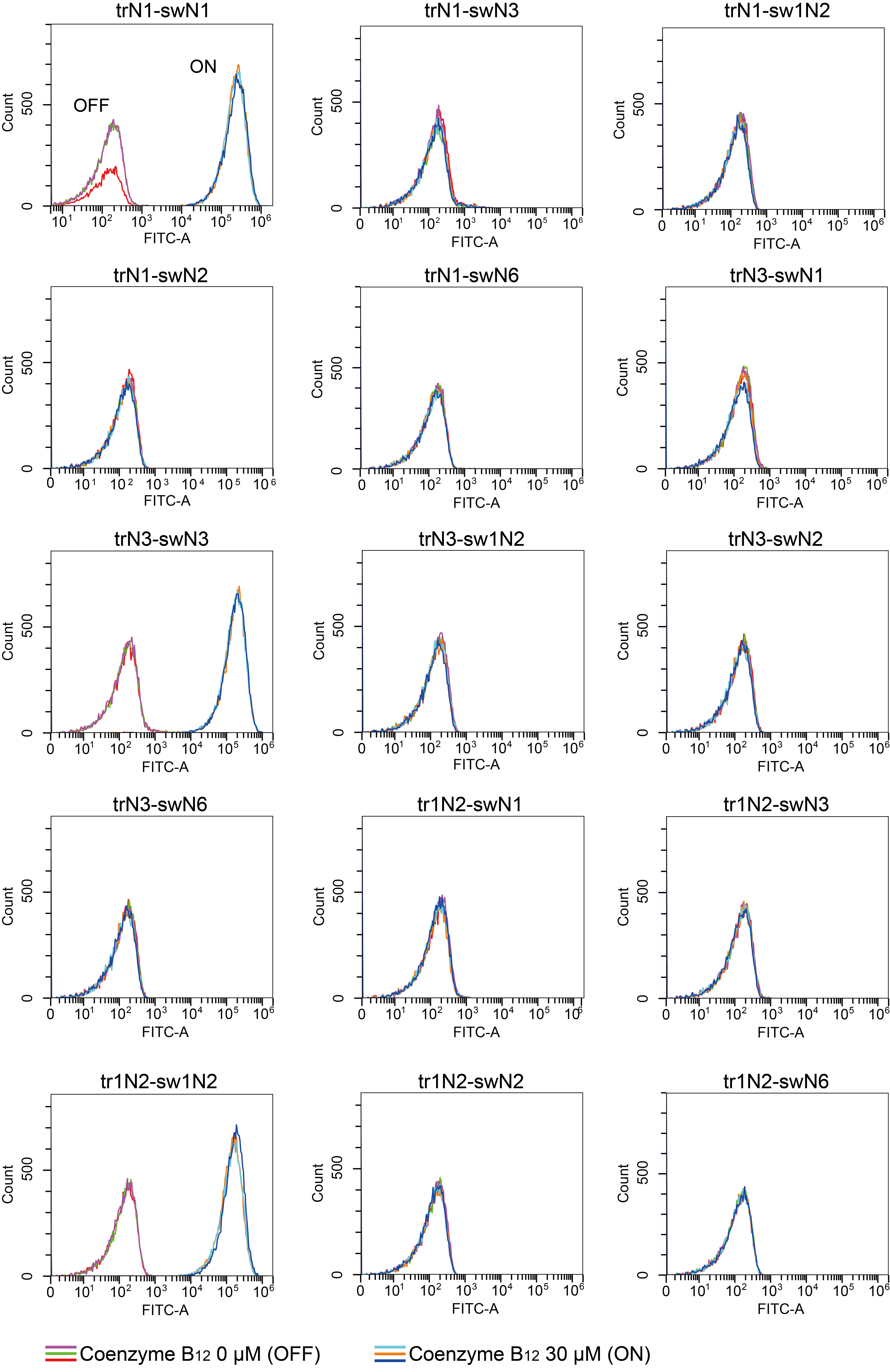

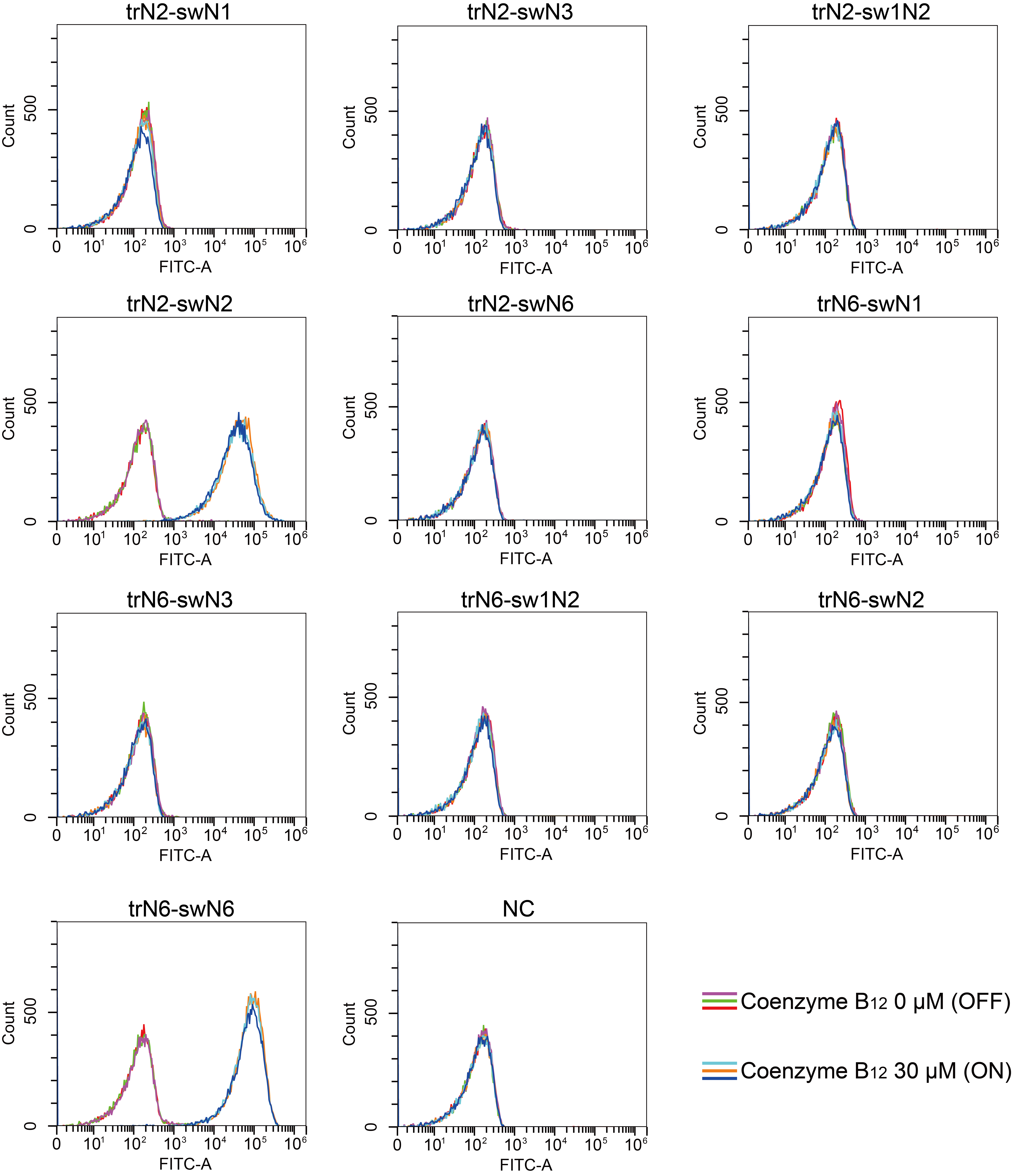


**Figure S3.** Histograms of the toehold switch-based modulators measured by flow cytometry. The fold-changes of average fluorescence in the presence and absence of coenzyme B_12_ are plotted in Figure 3b. The strain NC (negative control) refers to BL21 star (DE3) without toehold switch-based modulator.


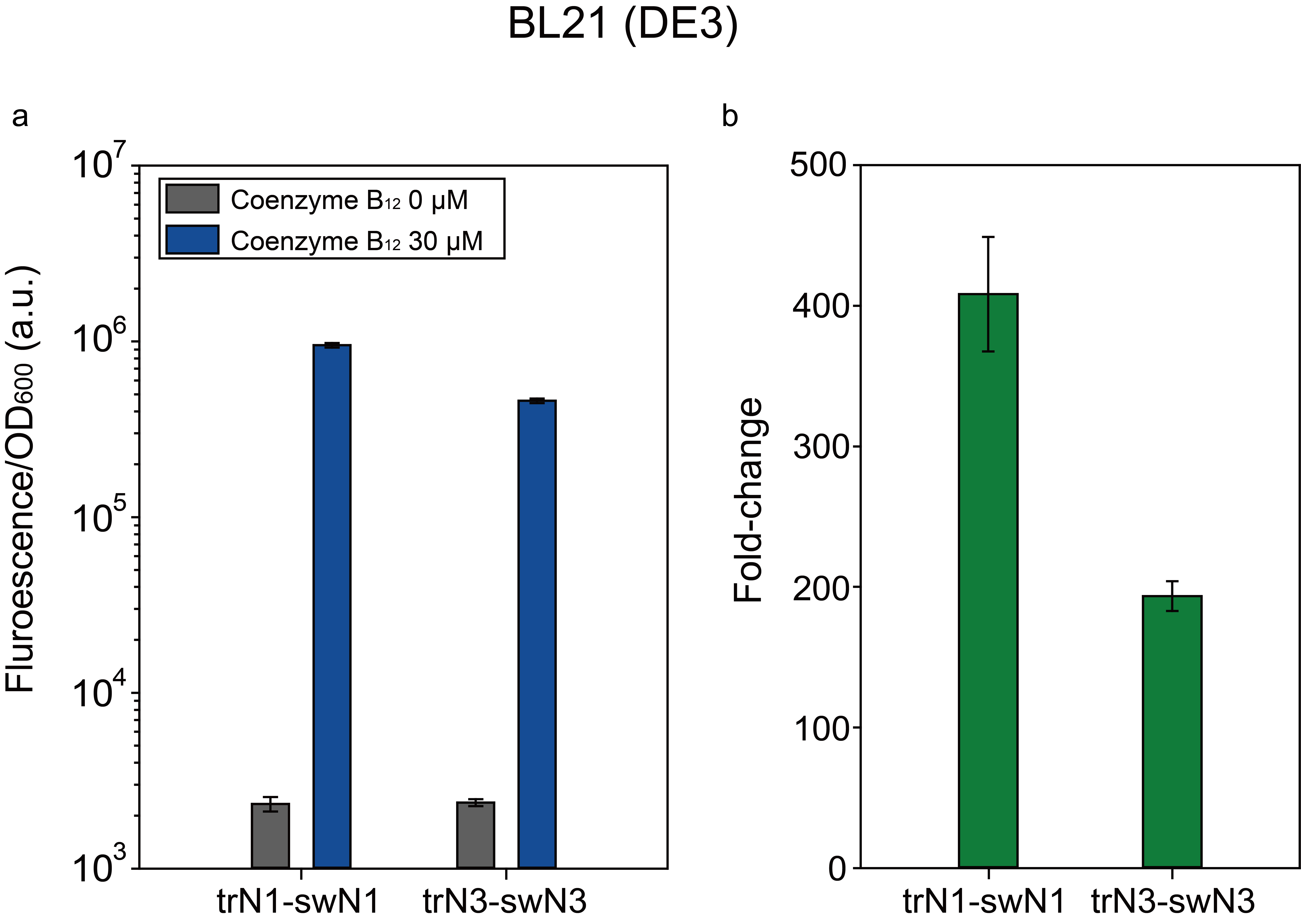


**Figure S4.** Performance of toehold switch-based modulator in *E. coli* BL21(DE3). (a) Fluorescence measurements for BL21-trN1-swN1 and BL21-trN3-swN3 with and without coenzyme B_12_ (30 μM). (b) Fold-change of GFP reporters for BL21-trN1-swN1 and BL21-trN3-swN3. Error bars indicate standard deviations from triplicate measurements.

**References**

Jang S, Jang S, Noh MH, Lim HG, Jung GY. Novel Hybrid Input Part Using Riboswitch and Transcriptional Repressor for Signal Inverting Amplifier. ACS Synth Biol. 2018;7:2199–204.

Green AA, Kim J, Ma D, Silver PA, Collins JJ, Yin P. Complex cellular logic computation using ribocomputing devices. Nature. 2017;548:117-121.
